# Precision of cementum annuli method for aging male white-tailed deer

**DOI:** 10.1371/journal.pone.0233421

**Published:** 2020-05-21

**Authors:** Daniel M. Adams, Julie A. Blanchong

**Affiliations:** Department of Natural Resource Ecology and Management, Iowa State University, Ames, Iowa, United States of America; Texas State University, UNITED STATES

## Abstract

The most common method used to estimate ages of harvested white-tailed deer (*Odocoileus virginianus*) and other cervids is a criterion based on tooth replacement-and-wear (TRW). Previous studies have shown this method is prone to considerable error because TRW is partially subjective. A presumably more accurate, but more labor intensive and expensive, method to estimate age involves the counting of cementum annuli (CA) of cross-sectioned incisors. Quantifying rate of error of the CA aging method is not possible without known-aged specimens, but precision of duplicate CA age estimates for two teeth may be related to accuracy if identical factors influence both CA accuracy and precision. The objective of this research was to identify and assess factors affecting precision of paired CA ages as well as evaluate congruence between TRW and CA age estimates. We obtained paired CA age estimates from a laboratory specializing in CA aging for 473 adult (≥ 1 year old), male white-tailed deer harvested in Iowa (USA; 2014–2018). Not all CA age estimates of paired incisors agreed with one another and probability of agreement between the paired CA ages decreased as the certainty level of the CA ages provided by the laboratory decreased and was dependent upon the batches in which they were aged by the laboratory. We also estimated the age of 1,292 adult, male deer using both TRW and CA methods and compared the congruence between the TRW and CA age estimates. Congruence rates of CA and TRW ages differed among age classes (80% congruence in yearling TRW age classification, 65% with 2-year-olds, 78% with ≥3-year-olds). Our results showed that CA aging is imperfect and that the certainty level is an important factor to consider with CA ages, as shown in previous research, as is the batch in which the teeth were aged. We also confirmed previous studies’ findings that CA and TRW ages for adult deer are not always congruent, particularly in age classes other than the yearling age class. Our results suggest managers are best served by using TRW to age adult deer as yearlings or ≥2-years-old. If additional age classes are required, CA aging is likely to be a better tool than TRW.

## Introduction

Estimating the age structure of a population is a key component of many wildlife management programs, as demographic parameters (e.g., survival and fecundity) that can be important to population modeling are typically age-specific [1–3]. Furthermore, age-specific prevalence rates of highly-transmissible diseases (e.g., chronic wasting disease) have been of recent concern [4,5]. The age structure of harvested animals is typically used to reconstruct a population’s age structure, especially in cervid populations [6,7]. Inaccurate estimates of a population’s age structure could lead to potential downstream impacts on demographic and epidemiological models [7,8].

The most common method used to estimate age of white-tailed deer (*Odocoileus virginianus*) and other cervids is the tooth replacement-and-wear (TRW) criterion. Severinghaus [9] developed the TRW aging criterion for white-tailed deer based on unique teeth eruptions at younger ages and the wear and degradation of the same premolars and molars of the lower jaw at older ages. Aging deer using the TRW method is popular among management agencies because it can be conducted quickly in the field at relatively little cost [10]. Previous studies have shown the TRW method is prone to considerable error because it is partially subjective, especially at older age classes [10–14]. Error in the TRW method at older ages has led to recommendations to implement TRW aging categories for adult white-tailed deer that bin older age classes together: either suggesting the binning of all deer ≥2 years of age together [10,11,14–16] or differentiating deer 2 years of age from deer ≥3 years of age [12,13,17], with both points-of-view suggesting an independent age class for deer 1 year of age (yearlings), since a unique tooth eruption pattern can often identify the age class [9].

A more labor-intensive and expensive, but more accurate, option for aging deer is cementum annuli (CA) aging, which involves the counting of annual rings, the CA, in the roots of cross-sectioned incisors. Seasonal rates of cementum deposition in the root tips that coincide with body growth produce alternating light and dark bands, with the dark bands representing the CA [18,19]. The CA method has been established as a more accurate method than TRW through the comparison of known-age individuals, especially at northern latitudes [11,13,20]. While CA is more accurate than TRW, with the exception of a sample of 97 known-aged deer in Wisconsin that were aged 100% accurately by the CA method [15], error has been documented with CA aging in relation to known-age individuals with error rates ranging from 15–28% in northern latitudes [11,13,20]. While assessing error rates of CA age estimates in comparison to known-age individuals is the only method to identify the accuracy of CA aging for certain, obtaining known-age individuals is difficult outside of captive facilities and capture-recapture studies. In response to the challenges of obtaining actual accuracy rates of CA aging, evaluating the precision between the CA age estimates of multiple incisors from the same deer is an option for samples from wild populations of unknown ages because if factors known to affect CA accuracy can also be shown to be related to the precision of CA aging, the measurement of precision can provide insight related to accuracy of the CA method [10]. Few studies, however, have evaluated the precision of CA ages in white-tailed deer [10,11,21]. While Roseberry [11] and Storm et al. [10] extracted paired incisors from individual deer after harvest, DeYoung [21] extracted separate incisors one or two years apart from live deer. Precision of CA age estimates has also been examined in other cervids such as mule deer (*Odocoileus hemionus*; [22]) and moose (*Alces alces*; [23]). Storm et al. [10] and Asmus and Weckerly [22] examined factors influencing the precision of paired CA ages. Factors included the sex of the individual, precipitation during the individual’s life, the level of certainty assigned to the CA age estimates, as well as the age of the CA age estimates [10,22].

Managers must make decisions on how best to use limited resources when deciding on what methodology to use to age deer. Additional information is needed to help resolve whether, excluding fawns, two (yearling, ≥2 years of age) or three (yearling, 2 years of age, ≥3 years of age) age categories should be used if managers decide to rely on TRW for aging. While the accuracy of CA aging has been thoroughly investigated, further evaluation of factors affecting the precision of CA aging is warranted to provide guidance to managers on what factors they must consider when evaluating CA aging data. To that end, the first objective of this study was to evaluate the precision of the CA aging method of paired incisors from wild male white-tailed deer in Iowa. We hypothesized that precision would decrease as the age of the CA age estimate increased, the level of certainty of the estimate decreased, and that precision would be dependent upon the batches in which the teeth were aged, an *a posteriori* consideration after viewing the CA data. Our second objective was to evaluate factors influencing the level of certainty assigned to CA age estimates. We hypothesized that the level of certainty in the age estimates would decrease with age and be influenced by the batch in which the age estimates were aged. Our third objective was to assess congruence between the age estimates from the CA and TRW methods specifically when deer aged using TRW were aged exclusively as yearling, 2 years of age, or ≥3 years of age. We hypothesized that congruence between the two aging methods would be greatest within the yearling age class and lower for both of the older age classes because the TRW criteria within the yearling age class relies on the presence or absence of a tooth while older ages are determined by a subjective assessment of tooth wear.

## Methods

Staff from the Iowa Department of Natural Resources (DNR), Iowa State University (ISU) technicians, and ourselves extracted incisors as well as assigned ages, using the TRW method, to antlered, male deer that were harvested from 2014–2018 in Iowa, USA. We submitted I1, and rarely I2, incisors to Matson’s Lab (Manhattan, MT) for age estimation, to the year of age, by the CA method. The I1 incisors are the standard teeth preferred to be aged by Matson’s Lab for ungulates but aging of I2 incisors is possible when the lab is made aware (Matson’s Lab, unpublished cementum annuli age report). Matson’s Lab assigned each age estimate a certainty code describing the lab’s certainty in the accuracy of the estimate ranging from greatest (“A”) to least certainty (“C”). Certainty codes are subjective because assignment of them to an age is based on the similarity of cementum characteristics of the individual tooth with the standardized model used by the lab (e.g., “A” certainty code indicates near identical matches between histological evidence of tooth and standardized model, “B” indicates close resemblance, “C” indicates a poor match; Matson’s Lab, unpublished cementum annuli age report). For a subset of the deer aged, both I1 incisors were removed and submitted independently with unique identification numbers so Matson’s Lab was unaware they were aging two teeth from the same deer. We submitted four batches of incisors to Matson’s Lab in total (one after the 2016 deer hunting season, two after the 2017 season, one after the 2018 season) and the number of teeth per batch ranged from 297–582. Consultation with the Iowa State University Institutional Animal Care and Use Committee (IACUC) prior to the initiation of this study determined that because the samples used for this project were collected from deceased hunter harvested deer, an IACUC protocol was not required.

Most deer were harvested during deer hunting seasons in late autumn (Oct–Dec), with some harvest occurring in other deer hunting seasons in September and January. Deer were assigned ages using the TRW method in the field and at deer meat processors. Additional mandibles were also obtained from taxidermists and were assigned ages in a laboratory setting. For TRW aging, all individuals aging deer assigned animals to one of three age classes: yearling (1 year of age), 2 years of age, ≥3 years of age. Deer were harvested approximately six months between birth days, so the age classes represented age at the time of their last birthday. According to the TRW aging criterion [9], yearlings were identified by the presence of a deciduous tricuspid P4 premolar or, if the tricuspid premolar had already been lost, the emergence of the permanent, bicuspid P4 premolar that was relatively unstained when compared to other cheek teeth. By 2 years of age, deer contain all permanent teeth and age classes are distinguished by the amount of tooth wear and degradation (e.g., exposed dentine increases with tooth degradation and age). Deer at 2 years of age were distinguished from older deer by the lack of exposed dentine and wear on the lingual crests of the M1 molar and by identifying a slope towards the lingual side of the jaw by the posterior cusp of the M3 molar. We assumed that rates of tooth replacement and wear were consistent in deer within the sample. For CA aging, deer were assigned a year of age (e.g., yearling, 2, 3, 4, 5, etc.). In the analyses described below, older CA age estimates were grouped into ≥3 years of age only when comparing CA age estimates to TRW age estimates. For all other analyses involving CA age data, older aged deer were not grouped together.

As indicated above, incisors were submitted for aging in several batches. Preliminary exploration of the data indicated lower congruence between TRW and CA age estimates of yearlings from the first batch of teeth sent to Matson’s Lab compared to the second and third batch (i.e., batches for which we had paired ages; Table 1) and also lower than reported in the literature (e.g., [10]). Further, a higher percentage of paired CA ages agreed when a pair did not contain a tooth aged in the first batch than paired CA ages that did contain a tooth aged in the first batch. We suspected that there might be greater error in the CA age estimates in the first batch than in the second and third batches and that these preliminary findings were caused by human-associated (i.e., cementum laboratory employee examining teeth) error. Based on these preliminary findings, we used logistic regression to investigate the relationship between probability of agreement with models containing a two, three, or four-category batch letter coding system. To determine the impact of batch on probability of agreement between paired incisors we began by coding each pair of incisors with an identifying code indicating in what batch each tooth of the pair was aged. For example, if one tooth was aged in the first batch and the paired tooth was aged in the second batch, we assigned it code “1–2”. The remaining letter codes included “2–3”, “1–3”, and “3–3”. The fourth batch is not included in these classifications because none of the paired incisors were aged in that batch.

**Table 1:** Probability of Cementum Annuli (CA) age estimate matching tooth replacement-and-wear (TRW) age estimate for TRW age classes of harvested male deer in Iowa, USA, 2014–2018, based on different batches of incisors aged by CA Matson’s Lab.

	Estimated Age Class		
	1	2	≥3
Batch from Matson’s Lab			
**1**	0.74	0.72	0.83
**2**	0.89	0.54	0.78
**3**	0.85	0.57	0.79
**4**	0.77	0.68	0.76

Similar to Storm et al. [10], we also used logistic regression to assess the relationship between CA age estimate and batch code (based on the best model determined above) on the probability of at least one of the paired ages having a lower certainty code (i.e., not “A”). Age estimates with lower certainty codes of “B” or “C” were combined because there were only two “C” code teeth in the dataset.

We used logistic regression to evaluate factors influencing the probability of agreement of paired CA ages [10]. Our predictor variables were CA age estimate, certainty code (“A” vs. not “A”), and batch code (based on the best model determined above). For all regression analyses described here, we used Akaike’s Information Criterion corrected for small sample size (AIC_c_; [24]) to identify the best models. Models within 2 AIC_c_ of the highest-ranking model were considered to have equivalent support [24]. We also produced a cross-classification table illustrating probabilities of agreement between the paired CA age estimates. We estimated the probability of receiving a CA age estimate from a second aged incisor, given the age of the initial paired CA age estimate by assembling a cross-classification table of cell counts of each classification possibility, then dividing each cell count by the marginal total of the age class of the corresponding initial CA age estimate.

Lastly, we produced a cross-classification table of the probabilities of receiving a CA age estimate, given its corresponding TRW age estimate to assess congruence between the CA and TRW methods [10]. Before estimating probabilities, we binned CA ages into age classes identical to the TRW age classes to produce a symmetric table. We estimated probabilities of receiving a CA age estimate, given its corresponding TRW age estimate by dividing cell counts by the marginal total of the corresponding TRW age class. In order to include deer that had paired ages in this analysis, we assigned the first CA age estimate received from Matson’s Lab as the CA age estimate based on the assumption that managers using the CA method to age deer will typically have only one incisor aged by a lab.

## Results

We obtained TRW and CA age-estimates for 1,292 deer, including paired CA ages from 473 deer. The models evaluating the effect of the batches on agreement between the paired ages data were all within 2 AIC_c_ units (Table 2). The model containing the two-category batch variable differentiating between pairs that included an age from the first batch and pairs that did not was the top-ranked model (Table 2). The batch effect in this model, however, was not statistically significantly related to the probability of the paired CA age estimates being assigned the same age (β_batch_ = 0.38, *P* = 0.11, OR_batch_ = 1.47, 95% CI = 0.92–2.35). The only statistically significant effect in any of the three models was in the four-category model where batch code “2–3” significantly increased the probability of the paired CA age estimates being assigned the same age compared to batch letter code “1–2”. Since, according to AIC_c_, the models were considered equal, we chose to use the simplest, two-category batch variable in subsequent analyses.

**Table 2:** Summary of three models for analysis of influence of batch coding of paired Cementum Annuli (CA) ages on probability of agreement between paired CA ages of male white-tailed deer in Iowa, USA, 2014–2018.

Model	Δ AIC_c_^a^	*ω*_i_^b^
**Prob. of Agreement ~ Two-Category Batch Variable**^c^	0.00	0.44
**Prob. of Agreement ~ Four-Category Batch Variable**^d^	0.75	0.30
**Prob. of Agreement ~ Three-Category Batch Variable**^e^	1.01	0.26

Models are ordered by the difference between sample-size corrected Akaike Information Criterion of a particular model and the best model (Δ AIC_c_).

^a^AIC_c_ = 452.96

^b^*ω*_i_: Akaike weight

^c^Two categories: 1) Paired batches 1–2 & 1–3; 2) Paired batches 2–3 & 3–3

^d^Four categories: 1) Paired batch 1–2; 2) Paired batch 1–3; 3) Paired batch 2–3; 4) Paired batch 3–3

^e^Three categories: 1) Paired batches 1–2 & 1–3; 2) Paired batch 2–3; 3) Paired batch 3–3

The number of paired ages that contained at least one tooth with a certainty code that was not “A” was 104 of 473 (22%). The highest-ranking model describing the probability of at least one tooth from a deer receiving a certainty code that was not “A” contained only an age effect, and the model containing both an age and batch effect was within 2 AIC_c_ units. In the model containing only the age effect, the probability of at least one tooth receiving a certainty code that was not “A” increased with CA age (β_age_ = 0.24, *P* = 0.004, OR_age_ = 1.27, 95% CI = 1.08–1.49). In the model containing both an age and batch effect, the probability of at least one tooth from a deer receiving a certainty code that was not “A” increased with CA age but was not statistically related to batch.

**Fig 1:**
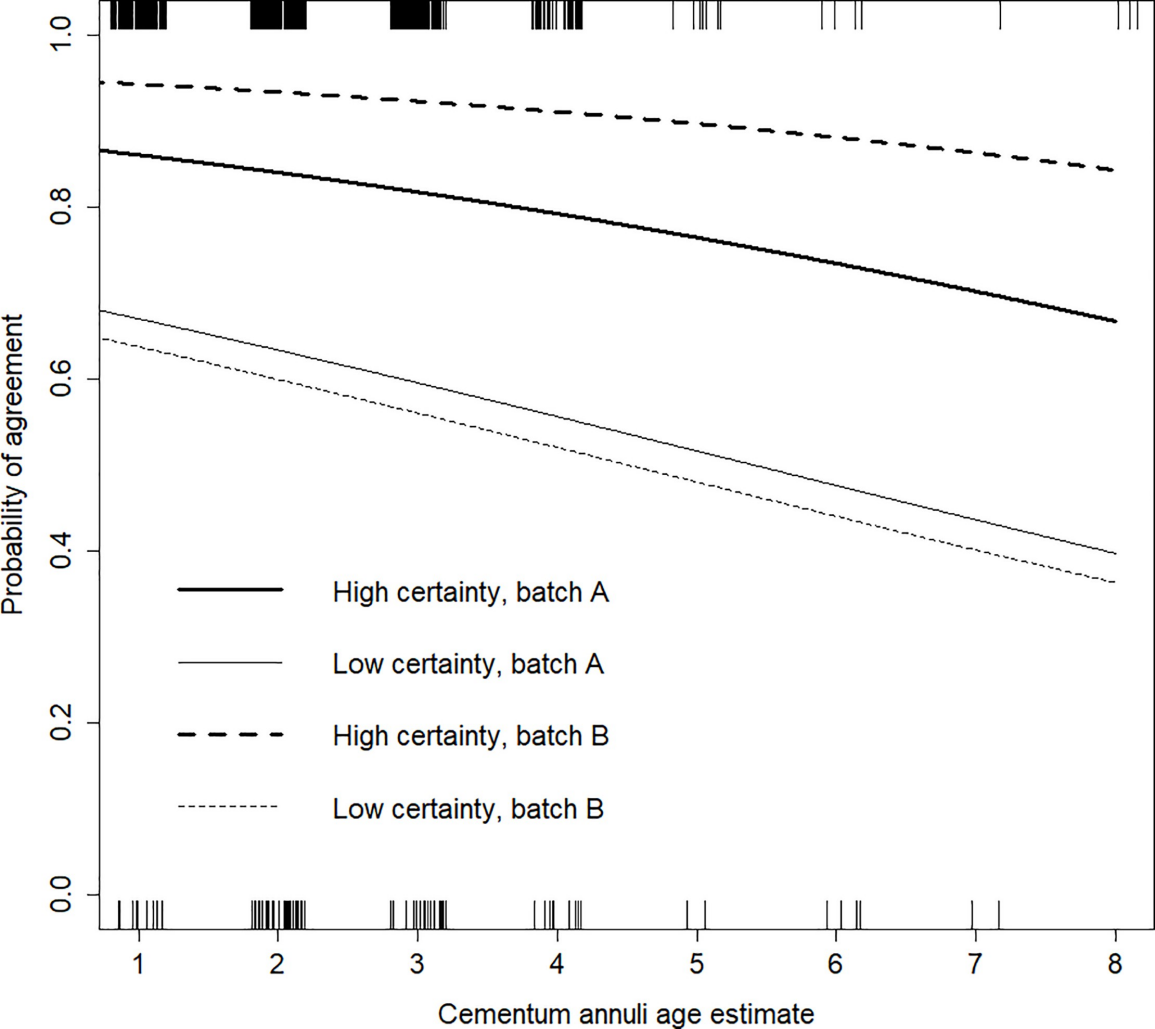
Predicted probability of agreement of paired Cementum Annuli (CA) age estimates of male white-tailed deer in Iowa, USA, 2014–2018, explained by CA age, Certainty Code (CC) of the age estimates, and the batch in which the paired incisors were aged.

Of the 473 paired CA ages, most (≈95%) were estimated in ages 1–4 and agreement between the first and second CA ages within those four ages ranged from 77–89%. Eighty-nine of the 473 paired ages disagreed (19%) but of the 89 that disagreed, 95% differed by only one year (two pairs differed by two years, two by three years, and one pair by four years). The top-ranked model describing probability of agreement between the paired CA ages contained the CA age, certainty code, and batch code terms, as well as an interaction between certainty code and batch. Two models were within 2 AIC_c_ units of the top model (Table 3). Both of these models contained an interaction between certainty code and batch code and one also had an interaction between certainty code and CA age. In the top-ranking model, probability of agreement was not statistically significantly related to age (β_age_ = −0.16, *P* = 0.09, OR_age_ = 0.85, 95% CI = 0.71–1.02), but was greater for high certainty teeth regardless of batch (Fig 1). Among high certainty teeth, agreement was greater for pairs in which neither of the paired ages were aged in the first batch sent to Matson’s Lab (Batch B) than for pairs in which one of the ages was aged in the first batch sent to Matson’s Lab (Batch A) (β_cc*batch_ = −1.13, *P* = 0.04). The interaction between certainty code and batch code was also statistically significant in the model containing an interaction between certainty code and CA age, which was not a significant interaction in that model.

**Table 3:** Summary of nine models for analysis of influence of Cementum Annuli (CA) ages, Certainty Code (CC), and batches in which the teeth were aged by matson’s lab on probability of agreement between paired CA ages of male white-tailed deer in Iowa, 2014–2018.

Model^a^	Δ AIC_c_^b^	*ω*_i_^c^
Prob. of Agreement ~ CA Age + CC*Batch	0.00	0.28
Prob. of Agreement ~ CC*Batch + CA Age*CC	0.50	0.22
Prob. of Agreement ~ CC*Batch	0.81	0.19
**Prob. of Agreement ~ CC + Batch**	2.44	0.08
**Prob. of Agreement ~ CA Age + CC + Batch**	2.45	0.08
Prob. of Agreement ~ Batch + CA Age*CC	3.02	0.06
Prob. of Agreement ~ Batch*CA Age*CC	3.56	0.05
**Prob. of Agreement ~ CA Age + CC**	4.60	0.03
**Prob. of Agreement ~ CA Age + Batch**	39.32	<0.01

Models are ordered by the difference between sample-size corrected Akaike Information Criterion of a particular model and the best model (Δ AIC_c_).

^a^If interactive effect is included in model, it is assumed additive effects of interaction are included as well.

^b^AIC_c_ = 410.14

^c^*ω*_i_: Akaike weight

“High certainty” denotes paired incisors that both received a CC of “A” and “low certainty” denotes paired incisors in which at least one age received a CC that was not “A”. “Batch A” denotes paired incisors where one of the age estimates was received from the first batch of incisors aged by Matson’s Lab within our dataset and “batch B” denotes paired incisors where neither of the age estimates were from the first batch from Matson’s Lab. Hash marks on the x-axes illustrate sample size of paired ages that agreed (top) and disagreed (bottom). Plot adapted from Storm et al. (2014).

The congruence between TRW and CA age estimates was greatest for yearlings and lowest for 2-year-olds (Table 4). For younger ages (i.e., 1 or 2 years of age), in the majority of instances of disagreement, the TRW age estimate was younger than the CA age estimate (Table 4).

**Table 4:** Probability of Cementum Annuli (CA) age class, given the tooth replacement-and-wear (TRW) age class for harvested male deer in Iowa, 2014–2018.

	CA Age Class				
	0	1	2	≥3	*n*
TRW Age Class					
**1**	<0.01	**0.80**	0.18	0.02	422
**2**	0.00	0.11	**0.65**	0.24	403
**≥3**	0.00	0.01	0.21	**0.78**	467

For deer where paired ages were available, the initial CA age was included. Cell values in bold indicate congruence of classifications between the CA and TRW methods. Sample size (*n*) indicates number of individuals placed within each TRW age class.

## Discussion

Precision of paired CA age estimates was influenced by an interaction between the level of certainty of the CA age estimate and the batches in which the CA age estimates were aged by Matson’s Lab. For high certainty teeth, specifically, the probability of agreement differed between pairs with one tooth aged in the first batch and pairs that did not include a tooth aged in the first batch. However, when at least one of the ages in a pair received a lower certainty code the batches the pairs were aged in was not important. Our results were consistent with findings of higher aging error rates for lower certainty teeth in a study of known-age deer [11] as well as studies with harvested deer [10,22]. Our results further suggest that certainty in the age estimate is a more important factor than batch on the probability that paired CA ages will agree. Therefore, managers receiving age estimates with certainty codes that are not “A” should consider having another incisor aged by CA or use the age cautiously, acknowledging a higher likelihood of error.

We were surprised that we failed to find a statistically significant association between CA age and CA precision. The mean effect of CA age that we observed (OR_age_ = 0.85) was nearly equal to that observed by Storm et al. (OR_age_ = 0.87; [10]). In Storm et al.’s [10] analysis, their data contained paired CA age estimates from both males and females that exceeded 15 years of age, whereas our dataset was limited to male deer up to 8 years of age and ≈95% of the deer were estimated at an age of 1–4 (24 out of 473 deer were estimated >4 years of age). It is possible that our dataset did not span a wide enough age distribution or did not contain enough samples at older ages to discern an effect of CA age on precision. While Hamlin et al. [13] observed proportionally more errors occurring in older deer (≥5 years old; 12 samples) than in deer that were younger (<5 years old; 62 samples), they were unable to detect an age effect on the accuracy of CA aging on known-aged white-tailed deer. Increased difficulty in aging older deer using CA has been noted due to annuli from older ages appearing very close together and less-distinguishable, apparently caused by a lessening in cementum production [19,25]. Incidence of these particular cementum characteristics possibly coincides with deer reaching asymptotic body size at 4–5 years of age [26,27], indicating peak maturity. Therefore, an effect of age on the probability of agreement between paired CA age estimates may only be apparent with deer of older ages, as CA become increasingly difficult to distinguish.

Matson’s Lab is the foremost histological lab specializing in cementum aging and has been used in many previous studies [10–13,21,22,28]. According to Matson’s Lab [29], typical accuracy of the CA method for white-tailed deer is 80–85% but is higher in deer from northern populations that are not supplementally-fed, which encompasses deer in Iowa. Inconsistencies within the cementum, such as the occurrence of split, compound, or false annuli or the thinning of annuli at older ages can affect the accuracy of CA age estimates [23,25] and may be related to the factors we explored in our study (e.g., age). The occurrence of these irregular structures is not consistent between paired incisors [30] and likely affects the precision of CA estimates as well as accuracy. Another factor influencing CA accuracy is human error, which can be related to the experience of the person counting the annuli or poorly-prepared incisor cross-sections [31]. The differences in precision related to different pairs of batches of incisors sent to Matson’s Lab that we observed may be at least partially explained by human error. Matson’s Lab was sold and moved to a new location in 2015 that possibly included personnel turnover, so the skill-level of observers may have improved from the first batch (aged in 2017) to the second and third batches (both aged in 2018). Other factors, such as age and certainty of a CA estimate, that have been shown to be related to the accuracy of CA aging [11,23,31] have also been demonstrated to influence the precision in CA aging [10,22]. The established impact of these factors on both accuracy and precision of the CA method has allowed researchers to draw inferences about CA accuracy from precision rates of paired CA ages [10,22].

Asmus and Weckerly [22] attempted to quantify error rates of the CA method using the precision of paired CA ages. They argued that since most paired CA ages that disagreed differed by only one year, it was likely that only one of the CA age estimates was incorrect, so the error rate of CA aging was half of the proportion of paired CA ages that disagreed (e.g., if 34% of paired CA ages disagreed, the error rate of CA was 17%; [22]). Following this methodology, since 19% of our paired CA ages disagreed, the observed error rate of CA aging would be 9.5%. Unfortunately, this approach could underestimate the error rate of CA aging when converting from precision of paired CA ages, as in some cases, neither age estimate may be accurate. Obtaining true accuracy rates of CA age estimates from the precision of paired age estimates alone is unlikely. Furthermore, Storm et al. [10] highlighted that precision, as well as accuracy, may be age- and sex-specific, rendering a single measurement of error uninformative.

Because CA age estimates are not always accurate, it is not possible to definitively evaluate accuracy of the TRW method using CA aging though comparisons between the two methods can be useful to identify the age classes for which each method might be strongest. The rates of congruence we found between the TRW and CA aging methods in the different age classes were similar to recent findings for deer in Illinois and Wisconsin [10]. We expected poorer congruence for older deer because the TRW method is generally not as accurate classifying older deer [12–14]. In addition, the low congruence in the 2-year-old age class may be related to the fact that the Iowa DNR uses a two-age classification system (yearling, ≥2 years of age) for adult deer (T. Harms, Iowa DNR, personal communication). As a result, Iowa DNR personnel may not have been as effective at distinguishing 2-year-olds from deer ≥3 years of age. The higher congruence of the ≥3-year-old age class than the 2-year-old age class likely was a result of the binning of multiple age classes into one, allowing for obviously older deer to be placed in this broad age class more accurately. As expected, we observed a high congruence between the two aging methods for the yearling age class likely because TRW is considered relatively accurate at that age as a unique, deciduous tooth or the recent eruption of the permanent tooth will often accurately identify yearlings [9]. However, the congruence for yearlings was not perfect. The observed incongruence could have been caused by personnel erroneously aging yearlings with a newly-erupted permanent tooth in late fall/early winter as an older deer because of the identification of the permanent tooth. The incongruence between aging methods in the yearling age class was also perhaps due to error within the CA age estimates, as we observed non-perfect precision with CA aging as well as congruence rates within the yearling age classes unexpectedly varying among batches aged by Matson’s Lab (Table 1). Studies that have examined the accuracy of the CA method for known-age yearlings have found some error in this age class. Using CA, 76% of a sample of 34 known-age white-tailed deer in Mississippi were aged correctly, whereas all 34 deer were aged correctly using TRW [12]. Hamlin et al. [13] had their only known-age yearling white-tailed deer in their sample aged incorrectly by CA (aged as 3-year-old with “B” certainty code) in Montana, although all seven of their yearling mule deer were aged correctly by CA.

## Conclusions

Due to the potential for misclassifications at older age classes with the TRW, our results suggest that managers that want to age deer using the TRW method should use a two-age classification system (yearling, ≥2 years of age) because the subjectivity of estimating older ages based on TRW can lead to bias in a reconstructed age structure [10,13,14,21]. The two-age classification system serves managers in scenarios where fecundity and survival rates are assumed to only differ between yearlings and deer ≥2-years-old [32]. If more age classes are desired, managers should utilize CA aging for deer ≥2 years of age. Managers should be aware that the level of certainty and batch of a CA age estimate influence the probability of agreement between paired CA ages, which are therefore also related to accuracy of CA age estimates. Options for dealing with lower certainty level CA age estimates include having another incisor from the individual deer aged, taking TRW age into consideration, or statistically-modeling the possibility of ±1-year-error in analyses as most errors have been demonstrated to be within one year of age.
